# Transient‐State Self‐Bipolarized Organic Frameworks of Single Aromatic Units for Natural Sunlight‐Driven Photosynthesis of H_2_O_2_

**DOI:** 10.1002/advs.202308322

**Published:** 2024-03-17

**Authors:** Wenjuan Zhang, Lizheng Chen, Ruping Niu, Zhuoyuan Ma, Kaikai Ba, Tengfeng Xie, Xuefeng Chu, Shujie Wu, Dayang Wang, Gang Liu

**Affiliations:** ^1^ State Key Laboratory of Inorganic Synthesis and Preparative Chemistry College of Chemistry Jilin University Changchun 130012 China; ^2^ Key Laboratory of Surface and Interface Chemistry of Jilin Province College of Chemistry Jilin University Changchun 130012 China; ^3^ Jilin Provincial Key Laboratory of Architectural Electricity & Comprehensive Energy Saving School of Electrical and Electronic Information Engineering Jilin Jianzhu University Changchun 130119 China

**Keywords:** H_2_O_2_ production, polymer photocatalyst, self‐polarization, solar energy conversion, through‐space π‐conjugation

## Abstract

Constructing π‐conjugated polymer structures through covalent bonds dominates the design of organic framework photocatalysts, which significantly depends on the selection of multiple donor‐acceptor building blocks to narrow the optical gap and increase the lifetimes of charge carriers. In this work, self‐bipolarized organic frameworks of single aromatic units are demonstrated as novel broad‐spectrum‐responsive photocatalysts for H_2_O_2_ production. The preparation of such photocatalysts is only to fix the aromatic units (such as 1,3,5‐triphenylbenzene) with alkane linkers in 3D space. Self‐bipolarized aromatic units can drive the H_2_O_2_ production from H_2_O and O_2_ under natural sunlight, wide pH ranges (3.0‐10.0) and natural water sources. Moreover, it can be extended to catalyze the oxidative coupling of amines. Experimental and theoretical investigation demonstrate that such a strategy obeys the mechanism of through‐space π‐conjugation, where the closely face‐to‐face overlapped aromatic rings permit the electron and energy transfer through the large‐area delocalization of the electron cloud under visible light irradiation. This work introduces a novel design concept for the development of organic photocatalysts, which will break the restriction of conventional through‐band π‐conjugation structure and will open a new way in the synthesis of organic photocatalysts.

## Graphical abstract

An entirely new strategy is developed to construct self‐bipolarized organic photocatalysts with single aromatic units. The resultant alkane‐linked organic frameworks can successfully drive the H_2_O_2_ production from H_2_O and O_2_ under natural sunlight irradiation.

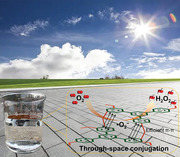

## 1 Introduction

Solar‐to‐chemical energy conversion for the production of high‐value chemicals via photocatalytic processes is a green and sustainable route to solve energy and environmental problems.^[^ ^1^, ^2^, ^3^, ^4^, ^5^ ^]^ In comparison with traditional inorganic semiconductors, organic frameworks photocatalysts have attracted great attention in recent years because the molecular backbone of organic frameworks offers a large chemical design space for improving optoelectronic and surface catalytic properties.^[^ ^6^, ^7^, ^8^, ^9^ ^]^ At present, the design of organic photocatalysts mainly originates from the principles and approaches of polymer synthesis, in which the well‐chosen monomers are jointed through covalent bonds to form a π‐conjugated chain‐like structure.^[^ ^10^, ^11^ ^]^ The classical regulation strategies include varying the oligomer length or the degree of polymerization and incorporating planar linkers to decrease the optical gap and/or prolong charge‐carrier lifetimes.^[^ ^12^, ^13^ ^]^ Most of recent work focused on the introduction of multiple donor‐acceptor building blocks into the polymer chain, in order to improve charge‐carrier delocalization and mobility.^[^ ^14^, ^15^, ^16^ ^]^ However, limited by the types of functional groups and their polymerization methods, few organic framework photocatalysts can achieve broad‐spectrum and/or natural sunlight‐driven photosynthesis.

In this work, we carried out an entirely new strategy to construct organic photocatalysts with single aromatic units based on the principle of through‐space conjugation.^[^ ^17^, ^18^, ^19^, ^20^, ^21^, ^22^ ^]^ The face‐to‐face overlapped aromatic units could be self‐bipolarized under light irradiation and drive the H_2_O_2_ production from H_2_O and O_2_ even under natural sunlight. Such a strategy could be achieved by simply alkane‐linking 1,3,5‐triphenylbenzene (TPB) in 3D space, which breaks the restriction of conventional through‐band π‐conjugation structure.^[^ ^23^, ^24^, ^25^ ^]^ The photocatalysts represent a broad‐spectrum response, and the optical properties and photocatalytic performance could be tuned by the length of alkane linkers. This novel design concept would pave the way for a new era in the development of organic photocatalysts.

## 2 Results and Discussion

### 2.1 Construction and Characterization of Photocatalysts

**Figure 1:**
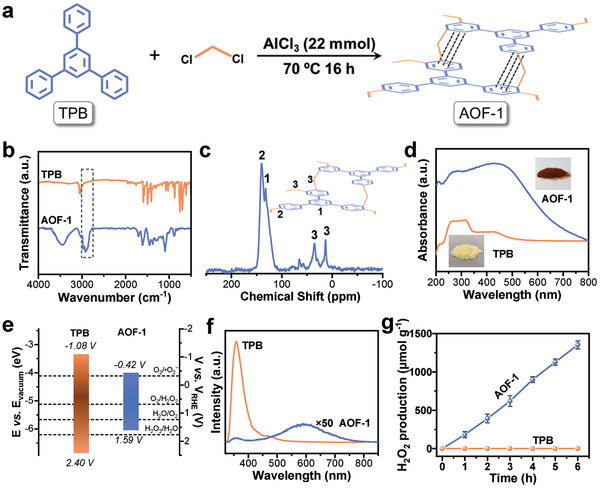
Structure and characterization of TPB and AOF‐1. a) Schematic illustration of the preparation of AOF‐1 by Friedel–Crafts alkylation reactions. b) FT‐IR spectra. c) Solid‐state ^13^C CP/MAS‐NMR spectra. d) UV–vis absorption diffuse reflectance spectra. e) Energy band diagrams. f) photoluminescence (PL) spectra using the excitation wavelength of 300 nm. g) Photocatalytic H_2_O_2_ production performance over AOF‐1 and TPB. Reaction temperature: 288 K, Xenon lamp (>420 nm).

The representative photocatalyst was first prepared by fixing TPB with CH_2_Cl_2_ via Friedel–Crafts reaction, leading to the formation of an alkyl (─CH_2_─) linked organic framework (denoted as AOF‐1, more details see in **Figure** **1**a and the Experimental Section). The Fourier transform infrared (FT‐IR) peaks at 2920–2960 cm^−1^ are ascribed to the strong C─H stretching vibrations of methylene groups (Figure 1b), which can be distinguished from ^13^C cross‐polarization/magic angle spinning nuclear magnetic resonance (^13^C CP/MAS NMR) spectroscopy (36 and 13 ppm).^[^ ^26^, ^27^ ^]^ The resonance peaks ≈140 and 131 ppm are assigned to the substituted aromatic carbon and unsubstituted aromatic carbon, respectively ^[^ ^28^, ^29^ ^]^ (Figure 1c). AOF‐1 possesses an amorphous microporous structure with a specific surface area of 2697 m^2^ g^−1^ and pore volume of 1.6 cm^3^ g^−1^ (Figures S1–S3, Supporting Information), which indicates TPB was fixed by methylene in 3D space to form AOF‐1.

UV−visible diffuse reflectance spectroscopy (UV–vis DRS) represents that TPB is a near‐ultraviolet‐response material, with an optical gap width of 3.48 eV (Figure 1d; Figure S4, Supporting Information). In sharp contrast, the absorption edge of AOF‐1 shows a significant redshift, approaching ≈800 nm. The corresponding optical gap width was narrowed to 2.01 eV (Figure 1d; Figure S4, Supporting Information). XPS valence band spectrum was adopted to determine the position of the highest occupied molecular orbital (HOMO). The work function of the XPS analyzer is ≈4.60 eV (vs vacuum). Therefore, combined with the valence band XPS spectrum (Figure S5, Supporting Information), the HOMO positions of TPB and AOF‐1 can be calculated as −6.84 and −6.03 eV. The lowest unoccupied molecular orbital (LUMO) positions are thus estimated at −3.36 and −4.02 eV. The electron volts are converted to electrochemical energy potentials in volts according to the reference standard for which 0 V versus RHE (reversible hydrogen electrode) equals −4.44 eV versus evac (vacuum level).^[^ ^30^, ^31^ ^]^ Thus, the HOMO positions of TPB and AOF‐1 versus RHE at pH 0 can be estimated at ≈+2.40 and +1.59 V (vs RHE at pH 0), respectively. The LUMO positions were calculated as −1.08 and −0.42 V (vs RHE at pH 0), respectively (Figure 1e).

In comparison with TPB, the clearly negative shift of HOMO and positive shift of LUMO of AOF‐1 indicate that the simple linkage of aromatic molecules through the methylene group greatly alters the optical properties, which are distinct from those of pristine monomers. The difference in optical properties of TPB and AOF‐1 was further analyzed with photoluminescence (PL) spectroscopy (Figure 1f). TPB exhibited a strong emission peak at 357 nm. This emission signal is very weak in AOF‐1. Instead, a new long‐wavelength peak at 600 nm appeared in the PL spectrum of AOF‐1. This result is consistent with the trend of changes in UV–vis DRS spectrum.

### 2.2 Photocatalytic Performance of AOF Photocatalysts

Photocatalytic conversion of earth‐abundant water and oxygen to H_2_O_2_ is one of the important ways of solar‐to‐chemical conversion (H_2_O+1/2 O_2_ → H_2_O_2_, ΔG^0^ = 117 kJ mol^−1^).^[^ ^32^, ^33^, ^34^ ^]^ As an environment‐friendly oxidant, H_2_O_2_ is widely used in organic synthesis and environmental remediation.^[^ ^35^, ^36^, ^37^, ^38^ ^]^ Under visible light (λ > 420 nm) irradiation, AOF‐1 could effectively drive the photosynthesis of H_2_O_2_ from H_2_O and O_2_ (Figure 1g). The production rate of H_2_O_2_ could be optimized to 2407 µmol g^−1^ h^−1^ (Figure S6, Supporting Information and the Experimental Section), which reaches the leading level in comparison with that of CTFs/COFs and C_3_N_4_‐based materials (Table S1, Supporting Information). In sharp contrast, TPB, the building unit of AOF‐1, does not show any activity in the mentioned reaction whether it is exposed to visible light or ultraviolet light (Figure 1g; Figure S7, Supporting Information). These results indicate that the linkage of aromatic molecules by the methylene group is a critical factor in constructing a visible‐light‐driven photocatalyst for H_2_O_2_ production.

**Figure 2:**
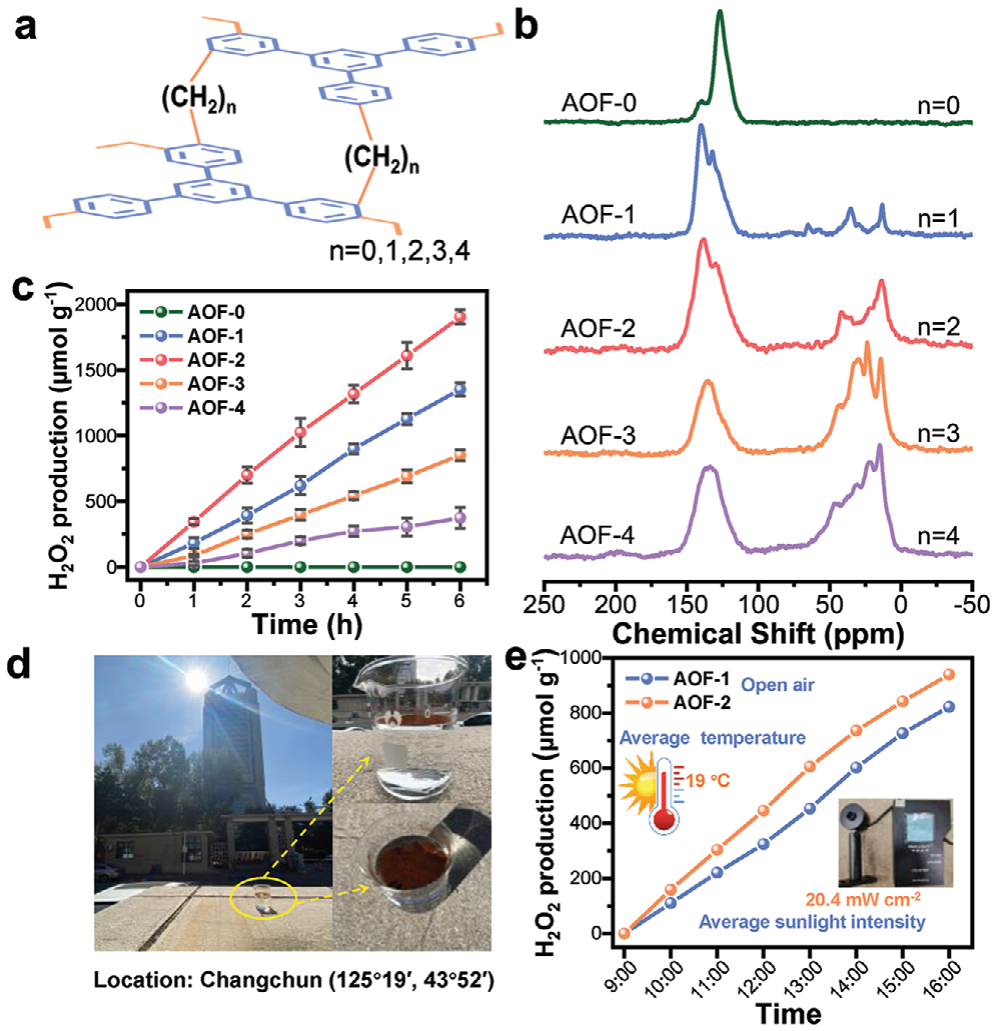
Structure and characterization of AOF‐n with different alkyl chain lengths. a) Schematic structures of different samples. b) Solid‐state ^13^C CP/MAS‐NMR spectra. c) Photocatalytic H_2_O_2_ production performance over different samples. d) Reaction setup for the natural sunlight‐driven photosynthesis of H_2_O_2_. e) Photocatalytic H_2_O_2_ production performance over AOF‐1 and AOF‐2 without stirring. The average temperature:19 °C and the average sunlight intensity: 20.4 mW cm^−2^.

The influence of the alkyl chain length on the optical properties and photocatalytic performance was further investigated. The alkyl chain was changed from ─CH_2_─to ─(CH_2_)_n_─ and the resultant materials are denoted as AOF‐n (*n* = 1–4) (**Figure** **2**a). The change of methylene signals in FT‐IR (at 2920–2960 cm^−1^) and ^13^C CP/MAS NMR spectroscopy (36 and 13 ppm) clearly confirms the increase of alkyl chain length in AOF materials (Figure 2b; Figures S8–S11, Supporting Information). The broad‐spectrum response and the suitable band structures for photocatalytic H_2_O_2_ production of all these samples were determined by UV–vis DRS (Figures S12–S14, Supporting Information), VB‐XPS (Figures S15 and S16, Supporting Information) and PL spectroscopy (Figure S17, Supporting Information). For comparison, another sample constructed by directly linking TPB molecules (denoted as AOF‐0) was synthesized via the Scholl reaction (Figure 2b; Figure S18, Supporting Information). AOF‐0 exhibits a much wider absorption range than other AOF samples (Figures S12–S14, Supporting Information), which should be due to the formation of traditional π‐conjugated structures in AOF‐0. The CB position (+0.08 V) of AOF‐0 is more positive than that of AOF‐n (*n* = 1–4), implying that the AOF‐0 may not be able to provide a sufficient thermodynamic driving force to facilitate an oxygen reduction reaction (Figures S15 and S16, Supporting Information). Electron paramagnetic resonance (EPR) shows that all samples exhibit a signal at g = 2.004, which could be assigned to the delocalized π‐electrons (Figure S19, Supporting Information). Transient photovoltage (SPV) spectra show that the TSC structure of AOF‐n (*n* = 1–4) facilitates charge transfer within the local surface area, as opposed to surface‐to‐bulk transport observed in AOF‐0 (Figure S20, Supporting Information). Finally, the performance of these materials on H_2_O_2_ photocatalytic synthesis was studied. It was found that AOF‐0 did not exhibit any photocatalytic activity in the production of H_2_O_2_ (Figure 2c). Meanwhile, AOF‐n (*n* = 1–4) samples are active for H_2_O_2_ production under visible light irradiation. Among them, AOF‐1 and AOF‐2 represent relatively high activity (Figure 2c).

**Figure 3:**
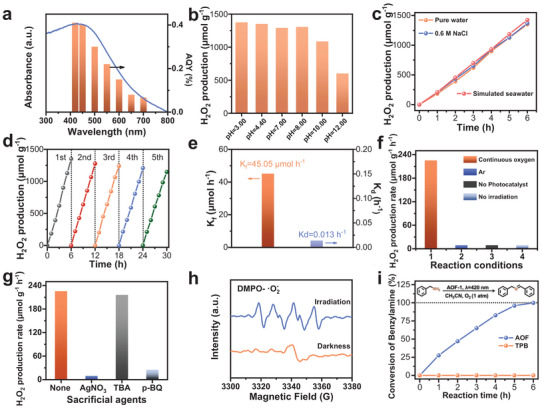
Photocatalytic performance. a) Wavelength‐dependent apparent quantum yield (AQY) measurement for AOF‐1. b) Photocatalytic H_2_O_2_ production over AOF‐1 in pure water at different pH. c) Photocatalytic H_2_O_2_ production for AOF‐1 in water containing different salts. d) Long‐term photocatalytic cycling experiments of AOF‐1. e) The H_2_O_2_ formation (K_f_) and the decomposition (K_d_) rate constant over AOF‐1. f) Photocatalytic H_2_O_2_ production rate under different conditions over AOF‐1. g) The investigation of the impact of additional scavengers on photocatalytic performance. h) EPR spectra of DMPO‐•O_2_^−^. i) Photocatalytic aerobic coupling of benzylamine on AOF‐1. Reaction conditions: substrate (1 mmol), catalyst (50 mg), O_2_ (1 atm), CH_3_CN (10 mL), UV–vis LED lamp (420 nm, 90 W), temperature (298 K).

The most ideal test to evaluate the activity of the sample is under the irradiation of natural sunlight, as the definite purpose of photocatalytic research is to achieve the conversion of solar energy to chemical products. In this case, the performance of AOF‐1 and AOF‐2 was studied under open air and natural sunlight irradiation (Figure 2d). A nearly linear growth can be observed from 9 am to 4 pm (Figure 2e), indicating AOF possesses excellent light harvest capability. We also measure the catalytic performance of AOF‐1 under different wavelength light irradiation. **Figure** **3**a shows that AOF‐1 has with broad‐spectrum response and can even achieve a photocatalytic synthesis of H_2_O_2_ under excitation at 700 nm. Also, such organic photocatalyst is applicable to a wide pH range (3.0–10.0) and a number of water sources including pure water, salt solution, simulated seawater, and natural resources like air as raw materials (Figure 3b,c). Moreover, AOF‐1 exhibits high stability in the photocatalytic reactions. It still maintains a linear increase after repeated five cycles of the test (Figure 3d).

The characterization results have shown that AOF‐1 meets the thermodynamic requirements for the photocatalytic synthesis of H_2_O_2_, where the levels of LUMO (−0.42 V vs RHE at pH 0) have a more negative potential compared to O_2_ reduction (+0.68 V vs RHE at pH 0), while the levels of HOMO (+1.59 V vs RHE at pH 0) have a potential correction relative to H_2_O oxidation (+1.23 V vs RHE at pH 0). Meanwhile, the AOF‐1 exhibits an ultralow H_2_O_2_ decomposition rate, which can effectively avoid the oxidative decomposition of H_2_O_2_ by the photo‐generated holes. (Figure 3e; Figure S21, Supporting Information).^[^ ^39^, ^40^, ^41^, ^42^ ^]^

The experimental evidence demonstrates that the production of H_2_O_2_ over AOF‐1 is a catalytic process initiated by the reduction of O_2_ under light irradiation (Figure S22, Supporting Information). First, almost no H_2_O_2_ can be detected in the absence of O_2_ or under dark conditions (Figure 3f). Second, trace amounts of H_2_O_2_ can be detected when an electron trapper (AgNO_3_) or an •O_2_^−^ scavenger (p‐benzoquinone, p‐Bq) is introduced in the reactions (Figure 3g). Third, the formation of •O_2_^−^ under light irradiation was measured by EPR measurements with 5,5‐dimethyl‐1pyrrolineN‐oxide (DMPO) as trapping agents (Figure 3h). In addition, the possibility of the formation of H_2_O_2_ via hydroxyl radical (•OH) can be ruled out because no DMPO‐•OH signal was measured in the dark or under light irradiation (Figure S23, Supporting Information). Figure 3g also shows that there is a negligible decrease in H_2_O_2_ production in the presence of •OH scavengers (t‐Butanol, TBA).

We also investigated the photocatalytic performance of AOF‐1 in oxidative coupling amines for imine synthesis. It is well‐known that imines are widely used in the biological and pharmaceutical area as reactive nitrogen‐containing compounds.^[^ ^43^, ^44^, ^45^ ^]^ The oxidative coupling of amines with air as the oxygen source is one of the most valuable routes for imine synthesis.^[^ ^46^, ^47^, ^48^ ^]^ In comparison with TPB, AOF‐1 shows relatively high activity in this reaction, which achieved 99.9% benzylamine conversion and 94.7% imine selectivity after a 6 h reaction. (Figure 3i). Moreover, a variety of amines containing electron‐donating (─OCH_3_, ─CH_3_) or electron‐withdrawing (─F, ─Cl, ─Br, ─CF_3_) groups can be catalyzed by AOF‐1 to produce the appropriate imines. A relatively low conversion was observed on a substrate of n‐butylamine, but the reaction showed a high selectivity to generate corresponding imines. (Table S2, Supporting Information). All these results show that AOF‐1 could be extended as an efficient catalyst for photocatalytic aerobic oxidation reactions.

To gain insights into the key intermediate involved in photocatalytic oxidation, a series of control experiments were conducted. No imine can be detected in the absence of O_2_, AOF‐1, and light, indicating that all the above factors were indispensable for efficient photocatalytic oxidation (Figure S24, Supporting Information). The conversion of benzylamine was decreased to 24.5% in the presence of p‐Bq as •O_2_^−^ scavenger, which indicated the vital role of •O_2_^−^ (Figure S25, Supporting Information). Moreover, by adding KI as a hole scavenger, a reduced conversion of 24.6% was obtained, suggesting that the photogenerated holes are important for activating amine molecules.

### 2.3 Insight Into the Impact of Alkane Linkage with DFT Calculation

Combining the characterization results of UV–vis DRS and PL spectroscopy and photocatalytic performance, it can be confirmed that AOF‐n (*n* = 1–4), due to the alkane linkage, possesses a different electronic structure in comparison with the TPB monomer. The most possibility is the formation of a through‐space conjugation structure between face‐to‐face overlapped aromatic rings of TPB, which is fully demonstrated in the subsequent theoretical investigation. Such structure not only narrows the optical gap of AOF‐n but also suppresses the combination of photo‐exited electrons and holes. In this process, the linked TPB units represent transient‐state self‐bipolarized states that impact the reactivity of AOF‐n in photocatalysis compared to the TPB monomer.

**Figure 4:**
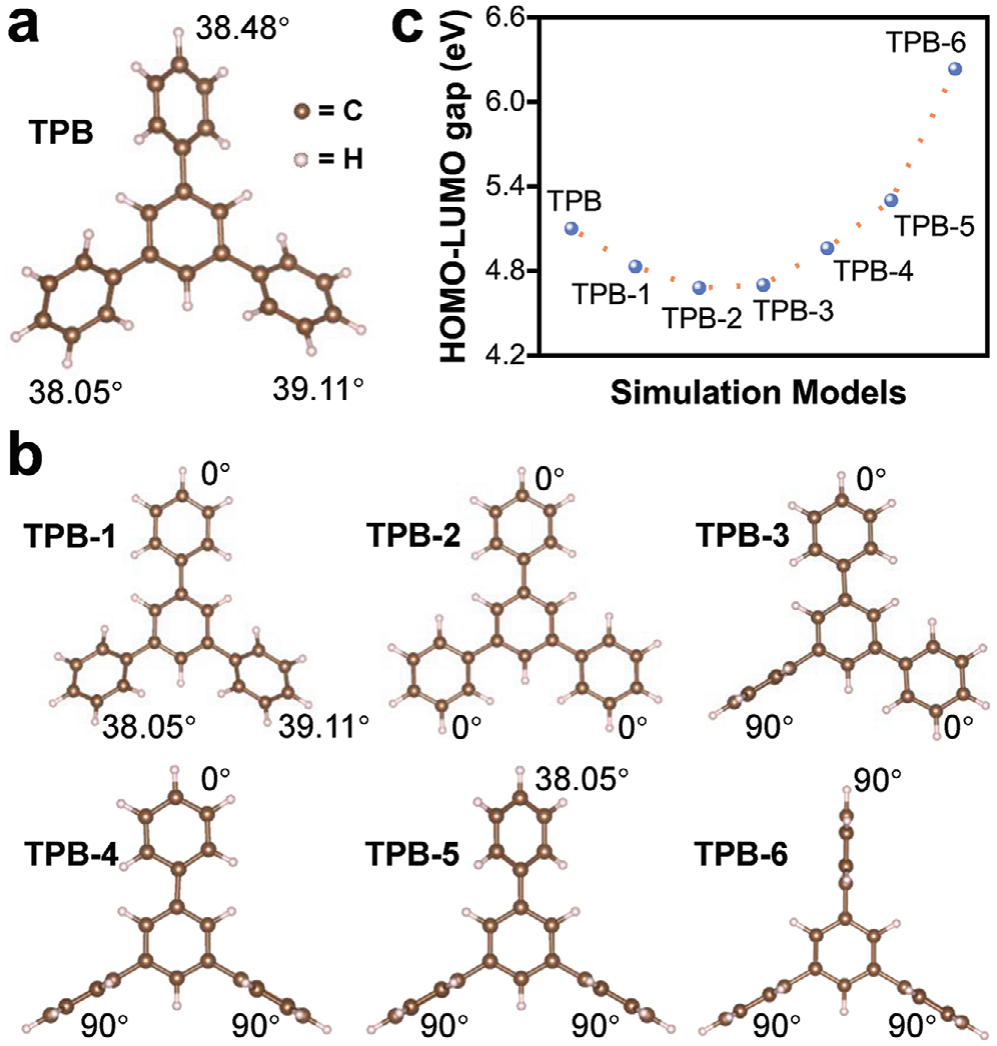
DFT calculations. a) The optimized structure of TPB molecule. b) The structure models and corresponding conformations of TPB molecules. c) The calculated HOMO–LUMO gap of TPB molecules with different conformations.

To confirm our speculations, DFT calculations were carried out to analyze the possible ways in which alkane linkers affect the structural properties of AOF‐n. First, we investigated the effect of alkane linkers on the conformation of TPB molecules. The TPB molecule is composed of four benzene rings that are linked in pairs through C─C σ bonds.^[^ ^49^, ^50^, ^51^ ^]^ Under thermodynamic stability, the three peripheral phenyl rings exhibit specific angles with the central benzene ring (**Figure** **4**a). After being linked by alkane groups, the angle between benzene rings can be altered to varying degrees, resulting in different conformations (Figure 4b; Table S3, Supporting Information). We calculated the HOMO–LUMO gap of these conformations, which changes from 5.10 to 4.68 eV (Figure 4c; Figure S26, Tables S3, and S4, Supporting Information). The small difference of 0.42 eV indicates that the conformation of the TPB molecule is not the determining factor that affects the optical properties.

**Figure 5:**
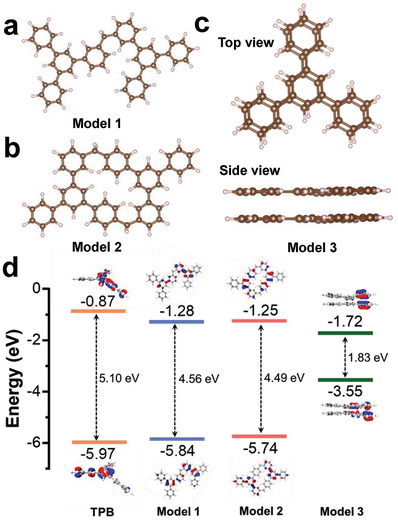
DFT calculations. a) The structure of Mode 1. b) The structure of Model 2. c) Top view and side view of the structure of Model 3. d) The main molecular orbitals of TPB molecules and stacked TPB dimer.

We further considered the scenario in which TPB is linked by alkane in a 2D plane. Within this scenario, there are two ideal models: one involving linear growth with TPB as the structural unit (**Figure** **5**a), and the other involving intermolecular closure into a ring (Figure 5b). After increasing the number of TPB molecules from two to six (Figure S27, Supporting Information), it was found that the HOMO‐LUMO gap width of the materials did not show a significant decrease (Figure 5d; Table S5, Supporting Information). Furthermore, there was only a slight difference of 0.07 eV between these two types of models. One possible explanation for this is that the use of alkane as the linking unit may block the through‐bond π‐coupling, leading to only a slight difference in the bandgap between the polymer and the monomer.

Significant changes in the HOMO–LUMO gap occurs in the scenario of vertical stacking of TPB molecules (Figure 5c). When the interlayer distances are 3.0 and 2.0 Å, the HOMO–LUMO gap widths decrease to 3.10 and 1.83 eV, respectively (Figure 5d). The HOMO–LUMO gap width of this simulated structure matches well with the measured results from the UV–vis DRS spectrum (2.01 eV). It gives strong evidence to support that the TPB molecules linked by alkane in the experiment formed the through‐space conjunction structure. This structure definitely narrows the optical gap and induces self‐bipolarization under visible light irradiation. To the best of our knowledge, this is the first time to design a polymer photocatalyst based on the principle of through‐space conjunction. Photocatalytic test and optical analysis fully prove that through‐space conjunction structure allows the efficient photoexcited electron and energy transfer between π‐electron systems, causing the natural sunlight‐driven photosynthesis of H_2_O_2_.

## 3 Conclusion 

Self‐bipolarized single aromatic units based on the mechanism of through‐space conjunction have been demonstrated to be efficient for the development of organic photocatalysts. This novel concept would significantly broaden the development of organic photocatalysts because it breaks the restriction of forming through‐band conjunction structure. What needs to be done is to spatially fix aromatic rings with alkane linkers of suitable length. The advantages of this strategy include 1) easily obtaining a broad‐spectrum response; 2) flexible selection of aromatic monomers. Our strategy also provides an opportunity for further improving the optical properties and photocatalytic performance through the introduction of electron acceptors or donors to the monomers and/or the 3D structure. The progress of this work would stimulate new thinking on the design of organic photocatalysts.

## Conflict of interest

The authors declare no conflict of interest.

## Supporting information

Supporting Information

## Data Availability

The data that support the findings of this study are available in the supplementary material of this article.
