# Supplementary material for: Transient‐State Self‐Bipolarized Organic Frameworks of Single Aromatic Units for Natural Sunlight‐Driven Photosynthesis of H2O2
Source: Adv Sci (Weinh). 2024 Mar 17;11(24):2308322. doi: 10.1002/advs.202308322 (PMC11200023; doi:10.1002/advs.202308322)
Supplement: Supplementary file 1 — Supporting Information [file ADVS-11-2308322-s001.pdf]

ADVANCED SCIENCE
Open Access

Supporting Information

for *Adv. Sci.*, DOI 10.1002/advs.202308322

Transient-State Self-Bipolarized Organic Frameworks of Single Aromatic Units for Natural Sunlight-Driven Photosynthesis of H_2O_2

*Wenjuan Zhang, Lizheng Chen, Ruping Niu, Zhuoyuan Ma, Kaikai Ba, Tengfeng Xie, Xuefeng Chu, Shujie Wu, Dayang Wang and Gang Liu**

Supporting Information

Transient-State Self-Bipolarized Organic Frameworks of Single Aromatic Units for Natural Sunlight-Driven Photosynthesis of H_2O_2

*Wenjuan Zhang, Lizheng Chen, Ruping Niu, Zhuoyuan Ma, Kaikai Ba, Tengfeng Xie, Xuefeng Chu, Shujie Wu, Dayang Wang, and Gang Liu**

W. J. Zhang, L. Z. Chen, R. P. Niu, Z. Y. Ma, D. Y. Wang, G. Liu
State Key Laboratory of Inorganic Synthesis and Preparative Chemistry, College of Chemistry
Jilin University
Changchun 130012, China
E-mail: lgang@jlu.edu.cn

W. J. Zhang, L. Z. Chen, R. P. Niu, K. K. Ba, T. F. Xie, S. J. Wu, G. Liu
Key Laboratory of Surface and Interface Chemistry of Jilin Province, College of Chemistry
Jilin University
Changchun 130012, China

X. F. Chu
Jilin Provincial Key Laboratory of Architectural Electricity & Comprehensive Energy Saving, School of Electrical and Electronic Information Engineering
Jilin Jianzhu University
Changchun 130119, China

Table of Contents

1. Experimental Procedures...S3
   1.1Reagents...S3
   1.2 Physical measurements..S3
   1.3 Preparation of alkane-linked organic frameworks............................S4
   1.4 Procedures for the photocatalytic H_2O_2 production..........................55
   1.5 Quantification methods..S6
   1.6 Photocatalytic H_2O_2 decomposition over AOF-1..............................S7
   1.7 H_2O_2 production performance on different condition.........................S7
   1.8 Cycling Experiments..S7
   1.9 Procedures for the oxidative coupling of amines...............................S7

1.10 Theoretical Calculations..S8
2. Characterization data...S8
Figure S1 X-ray diffraction (XRD) patterns..S8
Figure S2 N_2 adsorption–desorption isotherms of AOF-1.....................................S9
Figure S3 SEM and TEM images...S10
Figure S4 Tauc plot calculating the optical band gap..S11
Figure S5 Valence-band XPS spectra..S11
Figure S6 Photocatalytic H_2O_2 production performance over AOF-1..................S11
Table S1 Photocatalytic performance for H_2O_2 production from H_2O and O_2 in the reported systems..S12
Figure S7 Photocatalytic H_2O_2 production performance over TPB......................S13
Figure S8 X-ray diffraction (XRD) patterns of all samples.................................S13
Figure S9 FT-IR spectra of all samples..S14
Figure S10 SEM images of corresponding samples..S14
Figure S11 N_2 adsorption–desorption isotherms and pore-size distributions.......S15
Figure S12 Photographs of different samples..S15
Figure S13 UV–Vis diffuse reflectance spectra of different samples..................S15
Figure S14 Tauc plot calculating the optical band gap..S16
Figure S15 Valence-band XPS spectra..S16
Figure S16 Energy band diagrams of different samples......................................S16
Figure S17 PL emission spectra of different samples..S17
Figure S18 The possible structural of AOF-0 prepared by Scholl Reaction.........S17
Figure S19 EPR spectra of different samples..S18
Figure S20 TPV signal of different samples..S18
Figure S21 The photocatalytic decomposition of H_2O_2.......................................S19
Figure S22 Proposed H_2O_2 generation mechanism over AOF-1.........................S19
Figure S23 EPR spectra of DMPO-•OH for AOF-1..S19
Table S2 Photocatalytic aerobic coupling of different amines..............................S20
Figure S24 Photocatalytic oxidation activity of benzylamine.............................S21
Figure S25 Oxidative coupling of benzylamine with scavengers........................S21
Table S3 The calculated HOMO-LUMO gap of TPB molecules with different conformations...S21
Figure S26 The structure of methyl substituted TPB...S22

Table S4 The calculated bandgap of methyl substituted TPB with corresponding conformations..S22
Figure S27 The structure of planar trimers and hexamers formed through TPB polymerization..S23
Table S5 The calculated bandgap of different model...S23
3. References..S24

1. Experimental Procedures

1.1 Materials and reagents

1,3,5-triphenylbenzene (TPB), Aluminum chloride ($AlCl_3$, anhydrous), Dichloromethane (CH_2Cl_2), 1,2-Dichloroethane ($C_2H_4Cl_2$), 1,3-Dichloropropane ($C_3H_6Cl_2$) and 1,4-Dichlorobutane($C_4H_8Cl_2$) were obtained from Aladdin Reagent Co., Ltd. (Shanghai, China). Hydrochloric acid (HCl), ethanol (EtOH), methanol (MeOH) and tetrahydrofuran (THF) were purchased from Tianjin Guangfu Fine Chemical Research Institute (Tianjin, China). 30% H_2O_2 stock solution, nitric acid (HNO_3), sodium hydroxide (NaOH), potassium iodide (KI) and ammonium molybdate were obtained from Beijing Chemical Works. All solvents and reagents obtained from commercial sources were used without further purification.

1.2 Physical measurements

Solid-state NMR (^{13}C CP/MAS NMR) experiments were performed using a Bruker Avance III 400 MHz Solid-State NMR spectrometer at an external magnetic field of 9.4 T with a 4 mm doubletuned MAS probe at a spinning rate of 14 kHz. Fourier transform Infrared (FT-IR) spectra were measured on a Thermo Nicolet 6700 FT-IR spectrometer in the range of 4000-500 cm^{-1}. N_2 adsorption-desorption isotherms were obtained at 77 K using a Micromeritics ASAP 2010N analyzer. The samples were degassed at 473 K for 20 h prior to the measurements. Specific surface area measurements were obtained using the Brunauer-Emmett-Teller model within a pressure range of P/P_0 = 0.05−0.25. The pore-size distributions were evaluated using density functional theory calculations. Powder X-ray diffraction (XRD) patterns were recorded on a Rigaku X-ray diffractometer using Cu-Kα radiation over a 2θ range from 5° to 80°. Scanning electron microscopy (SEM) was performed using a Hitachi SU8020 instrument at 30 kV. Transmission electron microscopy (TEM) images were

obtained using an FEI Tecnai F20 EM with an accelerating voltage of 200 kV equipped with an energy-dispersive spectroscopy analyzer. X-ray photoelectron spectroscopy (XPS) measurements were performed on an X-ray photoelectron spectrometer (Thermo ESCALAB 250). UV-vis diffuse reflectance spectra were obtained from a UV-vis spectrophotometer (Shimadzu-3600) equipped with an integrating sphere and with $BaSO_4$ as a reference. The wavelength scanning range was 200-800 nm. Photoluminescence (PL) spectroscopy was carried out on the FLS920 (Edinburgh Instrument) at room temperature using the excitation wavelength of 400 nm. Transient photovoltage (TPV) measurement was carried out on the device provided by Xie's Groups [1]. Eelectron paramagnetic resonance (EPR) spectra were measured using a an ESR spectrometer (Bruker BioSpin, E500). 5,5-dimethyl-1-pyrroline N-oxide (DMPO) were used as the radical trapping reagent to detect •OH or •O_2^-. 2 mg catalyst was dispersed in ultrapure water (500 μL) or a H_2O/MeOH (1:9, 500 μL) mixture. Then add DMPO (0.1 mmol) to the above system. A Xe lamp (λ > 420 nm) was applied as the light source.

1.3 Preparation of alkane-linked organic frameworks

Preparation of AOF-1. The AOF-1 was prepared via $AlCl_3$-catalyzed Friedel–Crafts reaction. Chlorinated alkane (CH_2Cl_2) was used as a solvent to mix commercial aromatic precursors (1,3,5-triphenyl benzene, TPB) and a Lewis acid catalyst ($AlCl_3$). The polymerization reaction was carried out in a 250 mL two-neck flask with a condenser. Typically, the catalyst ($AlCl_3$, 22 mmol, 3.0 g) was added to a CH_2Cl_2 (100 mL) containing TPB (0.77 g, 2.5 mmol). The reaction system was then stirred for 16 hours at 70 °C. After cooling down to room temperature, the resulting black precipitate was quenched using ethanol, washed thrice with HCl-H_2O [2:1 (v/v)] and twice with ethanol to remove catalyst residues. Subsequently, the solid was washed three times with water, CH_2Cl_2, acetone and tetrahydrofuran respectively to remove the unreacted monomer. Further purification of product was carried out by Soxhlet extraction with ethanol for 12 h, tetrahydrofuran for 12 h, and CH_2Cl_2 for 12 h. The product was dried at 100 °C to give AOF-1. 1.08 g of reddish-brown powder was obtained (yield based on TPB is about 140 %).

Preparation of AOF fixed with alkyl chains of different lengths. The AOF-0 was prepared by mechanochemical route. In a typical procedure, the TPB (0.77 g) and iron(III) chloride (15 mmol, 2.43 g) was added to a 45 mL zirconia vessel with twelve

10 mm zirconia balls and twelve 2 mm zirconia balls. The samples were ball-milled at 400 rpm for 1 h. After milling, the product was filtered, washed with ethanol, and then purified by Soxhlet extraction with ethanol for 24 h. The purified sample was dried in vacuo at 100 °C overnight. AOF-2, AOF-3, and AOF-4 follow the preparation route of AOF-1. The catalyst ($AlCl_3$, 22 mmol, 3.0 g) was added to a solution of TPB (0.77 g, 2.5 mmol) in different solvents to yield AOF-2 (100 mL, 1,2-dichloroethane), AOF-3 (100 mL, 1,3-Dichloropropane) and AOF-4 (100 mL, 1,4-Dichlorobutane). The aftertreatment process of AOF-2 products is the same as AOF-1. The aftertreatment methods for AOF-3 and AOF-4 are as follows. The reaction mixture was quenched by adding 50 mL of ice water. The mixture was stirred for another 2 hours for the complete quenching of $AlCl_3$. Organic layer was extracted and neutralized with sodium bicarbonate solution until the evolution of gas stops. Any remaining salts were extracted and washed with 50 mL water for three times. The final organic layer was evaporated to recover from the solvent in a rotatory evaporator. The final product was dried at 100 °C.

1.4 Procedures for the photocatalytic H_2O_2 production

The photocatalytic H_2O_2 reactions were carried out in a flowing gas diffluent system. The evaluation device for the photocatalytic H_2O_2 production is shown in the following figure. Catalyst (200 mg) and deionized water (200 mL) was dispersed in a reaction cell made of Pyrex glass by a magnetic stirrer. The reaction temperature was maintained at 288 K by a temperature-controlled water bath (deviation, ±0.5 K). Prior to the photocatalytic tests, bubble O_2 into the suspension and stir the suspension in the dark for 30 min. The bottle was photoirradiated using a 300 W xenon lamp with a cutoff filter ($\lambda \geq 420$ nm, average intensity: 164 mW cm^{-2}) and O_2 was continuously bubbled into the bottle. After sampling every 1 h, solid-liquid separation procedure was carried out with a filter of 0.22 μm. The residue liquid was detected to the concentration of H_2O_2.

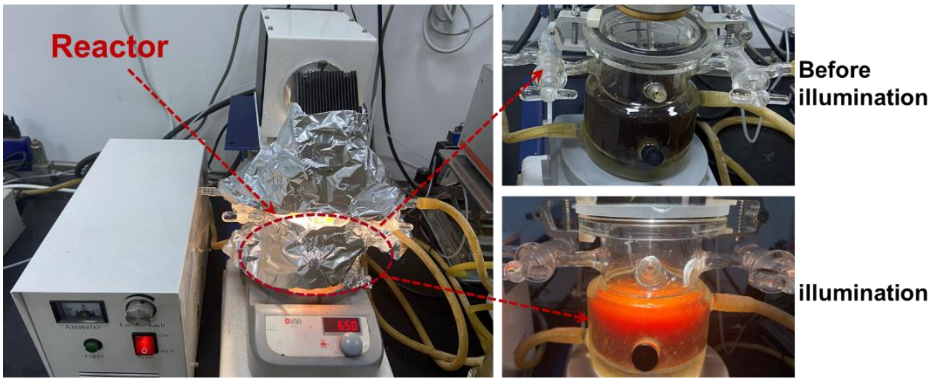

When optimizing the photocatalytic H_2O_2 performance, catalyst (5 mg) and deionized water (10 mL) was dispersed in a reaction cell by a magnetic stirrer. After sampling every 15 min, solid-liquid separation procedure was carried out with a filter of 0.22 μm.

For the photocatalytic H_2O_2 performance under direct natural sunlight, the catalyst (50 mg) was added to an beaker containing 200 mL of water, the reaction mixture under natural sunlight (in Changchun, China, during October 2023) and open air without stirring. The average sunlight intensity data was measured by a CEL-NP2000-10 with a photodiode sensor. After sampling every 1 h, solid-liquid separation procedure was carried out with a filter of 0.22 μm.

The apparent quantum yield (AQY) was measured under monochromatic light irradiation at a certain wavelength (λ =420, 450, 500, 550, 600, 650, 700 nm). The bandpass filters were used in measurement to obtained monochromatic light. The light intensity was measured by a CEL-NP2000-10 with a photodiode sensor. AQY at different wavelengths was calculated by the following equation:

$$AQY\% = \frac{\text{produced } H_2O_2 \text{ molecules (mol)} \times 2}{\text{incident photons number (mol)}} \times 100$$

$$= \frac{(\text{produced } H_2O_2 \text{ molecules})(N_A \times h \times c) \times 2}{I \times A \times t \times \lambda} \times 100$$

Where, N_a is Avogadro's constant (6.022×10^{23} mol^{-1}), h is the Planck constant (6.626×10^{-34} J s), c is the speed of light (3×10^{8} m s^{-1}), I is the intensity of irradiation light (W cm^{-2}), A is the irradiation area (cm^{2}), t is the photoreaction time (s), λ is the wavelength of the monochromatic light (m).

1.5 Quantification methods

To investigate the production trend of H_2O_2 during reaction, 2.0 mL of solution was collected after different reaction intervals for the detection of H_2O_2 concentration.

After filtered the photocatalysts, the concentration of H_2O_2 in the collected solution was detected by an iodometric method. Specifically, 2 M potassium iodide (1 mL) and 0.2 mM ammonium molybdate tetrahydrate (100.0 μL) were first added into the collected solution (2.0 ml). Then, these solutions were fully mixed and placed for 20 min. Finally, the color solution was detected by UV-vis spectrophotometry at 352 nm. The standard curve and equation of UV-vis absorbance were shown in the following figure.

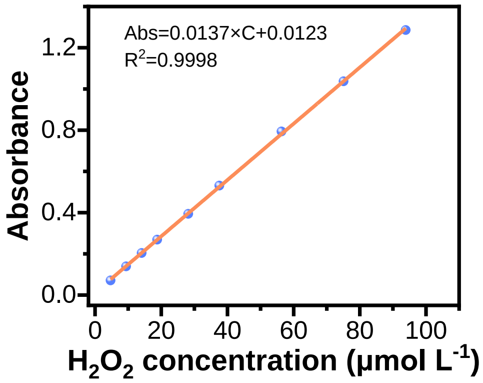

1.6 Photocatalytic H_2O_2 decomposition over AOF-1

Specifically, 200 mg AOF-1 were dispersed into 200 mL aqueous solution containing H_2O_2 (2 mM). Other reaction conditions were kept consistent with the photocatalytic H_2O_2 production except for the substitution O_2 with N_2.

1.7 H_2O_2 production performance on different condition

In order to explore the influence of pH on H_2O_2 production performance, 0.1 m HNO_3 or 0.1 m NaOH standard solution are used to adjust the pH of aqueous solution. In order to explore the influence of different sacrificial agents on the H_2O_2 production performance. The concentration of BQ and $AgNO_3$ added to the reaction system were both 1mM, and the concentration of TBA added to the reaction system was 2%. In order to explore the influence of different gases (N_2 or O_2) on the H_2O_2 production performance, N_2 was continuously injected into the reaction solution under dark conditions for 30 minutes to ensure that there was no residual O_2. After that, the photocatalysis test was carried out under the condition of continuous N_2 flow.

1.8 Cycling Experiments

After the photocatalytic reaction is completed, the AOF-1 was separated out by filtration. The solid was washed with distilled water, and then dried under vacuum at 100 °C for 12 h, yielding the AOF-1 for the next reaction sequence.

1.9 Procedures for the oxidative coupling of amines

In a typical amine oxidation reaction, a mixture of the catalyst (50 mg), benzylamine (100 μL), and acetonitrile (10 mL) was introduced into a reaction cell made of Pyrex glass. And the bottle was sealed with a rubber cap. After O_2 bubbling, the bottle was photoirradiated using a UV LED lamp (420 nm) in a temperature-controlled water bath (298K) with magnetic stirring. The products were collected *via* a sampling pipe and analyzed using a gas chromatograph equipped with an HP-5 column and an FID detector. The selectivity was determined based on imines and aldehyde as the products. The conversion of benzylamine and the selectivity of the imine for benzylamine were calculated using the area normalization method.

1.10 Theoretical Calculations

The Gaussian 09 software package is employed for density functional theory (DFT) calculations. The B3LYP hybrid functional was chosen as the computational method, and the 6-31G(d) basis set was used for the optimization and static calculations of the TPB unit to investigate the impact of TPB conformational changes on the electronic structure. To observe the spatial distribution of the highest occupied molecular orbital (HOMO) and lowest unoccupied molecular orbital (LUMO) on the molecular framework, an isosurface threshold of 0.05 was set to clearly visualize the electronic states.

2. Characterization data

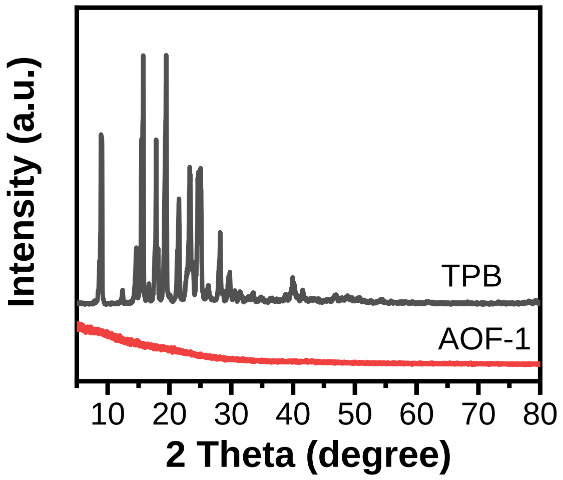

Figure S1. X-ray diffraction (XRD) patterns of TPB and AOF-1.

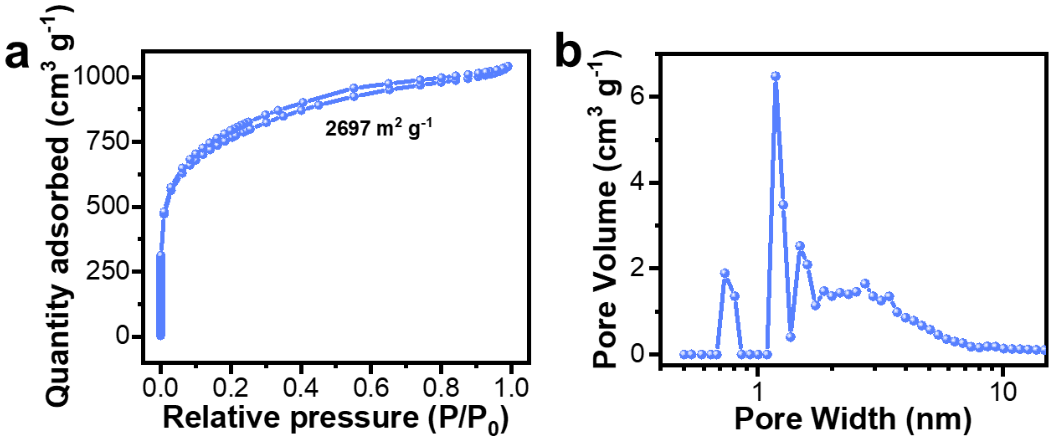

Figure S2. a) N_2 adsorption–desorption isotherms and b) pore-size distributions of AOF-1.

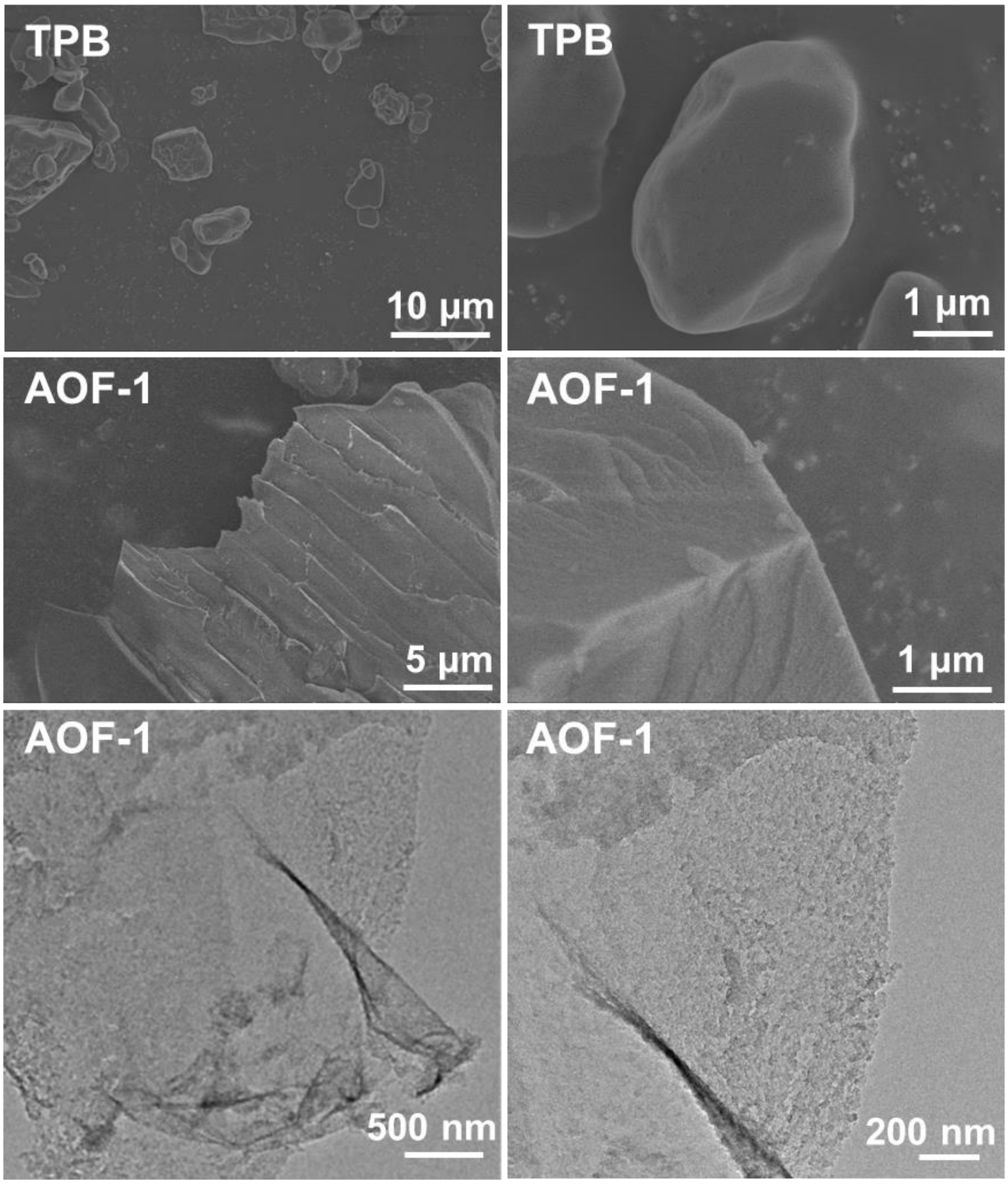

Figure S3. SEM images of TPB and AOF-1, and TEM images of AOF-1.

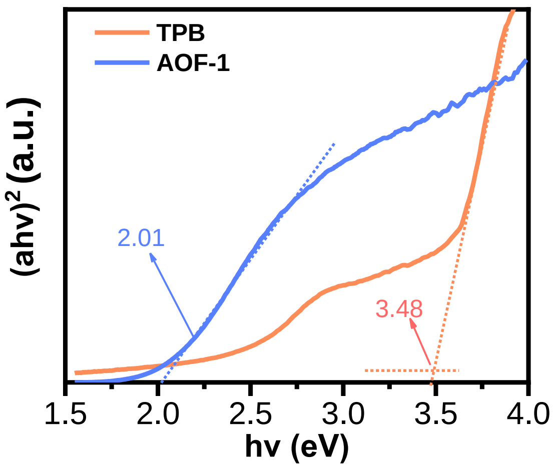

Figure S4. Tauc plot calculating the optical band gap of TPB and AOF-1.

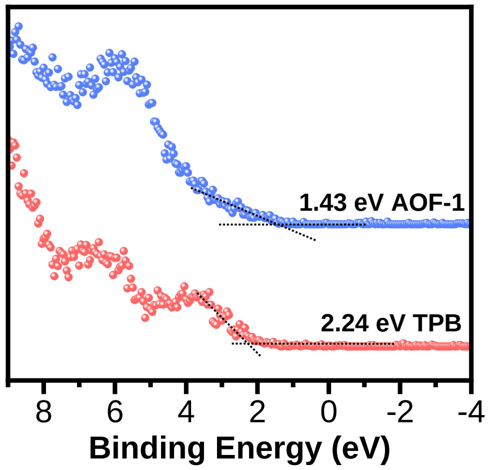

Figure S5. Valence-band XPS spectra of TPB and AOF-1.

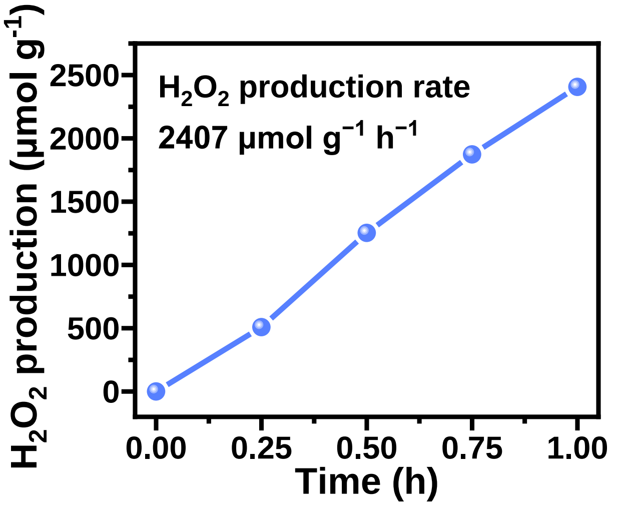

Figure S6. Photocatalytic H_2O_2 production performance over AOF-1. Reaction conditions: catalysts (5 mg), water (10 mL), temperature (288 K), Xenon lamp (> 420 nm).

Table S1. Photocatalytic performance for H_2O_2 production from water and O_2 in the reported systems.

Photocatalysts	Light source	Concentration of photocatalyst	Temperature (K)	rate of H_2O_2 formation/ μmol g^{-1} h^{-1}	Ref.
g-C_3N_4/PDI	Xe lamp (420–500 nm)	50 mg/30 mL	298	21	2
g-C_3N_4/BDI	AM1.5G simulated sunlight 100.0 W m^{-2} (λ>420 nm)	50 mg/30 mL	298	48	3
g-C_3N_4/PDI/rGO	AM1.5G simulated sunlight 100.0 W m^{-2} (λ>420 nm)	50 mg/30 mL	298	76	4
g-C_3N_4/MTI	AM1.5G simulated sunlight 100.0 W m^{-2} (λ>420 nm)	50 mg/30 mL	298	66	5
g- C_3N_4/PDI/BN/rGO	AM1.5G simulated sunlight 100.0 W m^{-2} (λ>420 nm)	50 mg/30 mL	298	104	6
RF Resins	AM1.5G simulated sunlight 100.0 W m^{-2} (λ>420 nm)	250 mg/50 mL	333	200	7
CTF-BDDBN	Xe lamp (λ>420 nm) 44.5 mW cm^{-2}	30 mg/50 mL	298	97	8
Co/AQ/C_3N_4	AM1.5G simulated sunlight 100 mW cm^{-2}	6 mg/12 mL	293	124	9
ZnPPC-NBCN	Xe lamp (λ > 400 nm) 100 mW cm^{-2}	10 mg/20 mL	298	114	10
RF P_3HT	AM1.5G simulated sunlight (λ>300 nm) 100 mW cm^{-2}	150 mg/50 mL	333	615	11
3DOM g-C_3N_4-PW11	Xe lamp (λ>420 nm)	100 mg/100 mL	298	24	12
R370-CN	Xe lamp (λ>420 nm)	100 mg/100 mL	298	170	13
TTF-BT-COF	Xe lamp (λ>420 nm)	5 mg/10 mL	298	2760	14
COFTfpBpy	xenon lamp (λ>420 nm) 40.8 mW cm^{-2}	15 mg/10 mL	298	694.7	15
NMT400	AM1.5G simulated sunlight	20 mg/50 mL	/	270.9	16
DETH-COF	Xe lamp (λ>420 nm)	10 mg/50 mL	298	1665	17
HEP-TAPT-COF	Xe lamp (λ>420 nm) 100 mW cm^{-2}	50 mg/100 mL	298	1750	18
RF-DHAQ	Xe lamp (λ>420 nm)	10 mg/50 mL	298	1820	19
BBTz	Xe lamp (λ>365 nm)	5 mg/ 25 mL	/	7274	20
DMCR-1NH	Xe lamp (λ>420 nm)	5 mg/11 mL	298	2588	21
TZ-COF	Xe lamp (λ>420 nm)	45 mg/30 mL	298	268	22
P-TAME	420 nm LED	20 mg/20mL	298	1900	23
TD-COF	white LED (400-700 nm) 100 mW cm^{-2}	1 mg/4 mL	/	4620	24
FS-COFs	Xe lamp (λ>420 nm)	5 mg/20 mL	/	3904	25
sonoCOF-F2	Xe lamp (λ>420 nm)	50 mg/ 60 mL	298	2736	26
TaptBtt	Xe lamp (λ>420 nm)	15 mg/ 10 mL	298	1407	27
TDB-COF	AM1.5G simulated sunlight	10 mg/10 mL	273	723.5	28
TpAQ-COF-12	Xe lamp (λ>420 nm)	10 mg/30 mL	/	420	29
Bpt-CTF	Xe lamp (350-780 nm)	10 mg/50 mL	/	3268.1	30
AOF-1	Xe lamp (λ>420 nm) 164 mW cm^{-2}	5 mg/10 mL	288	2407	This work

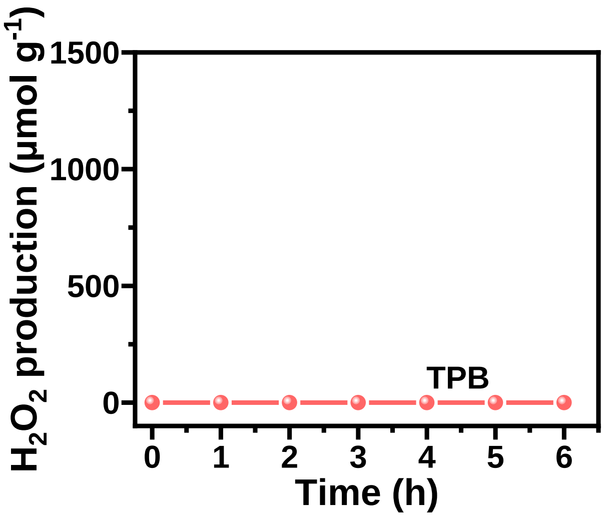

Figure S7. Photocatalytic H_2O_2 production performance over TPB using a 300 W Xenon lamp without a filter (full spectrum).

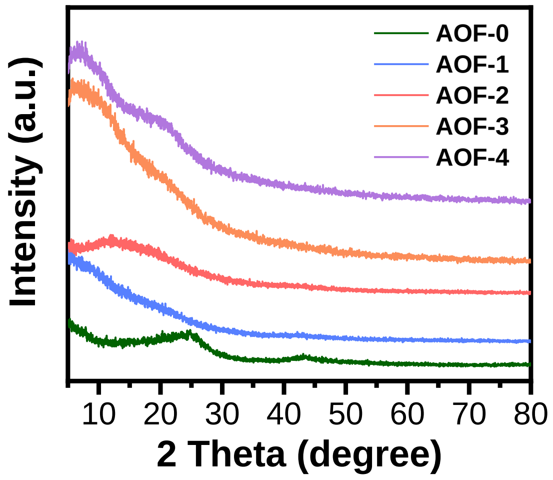

Figure S8. X-ray diffraction (XRD) patterns of different samples.

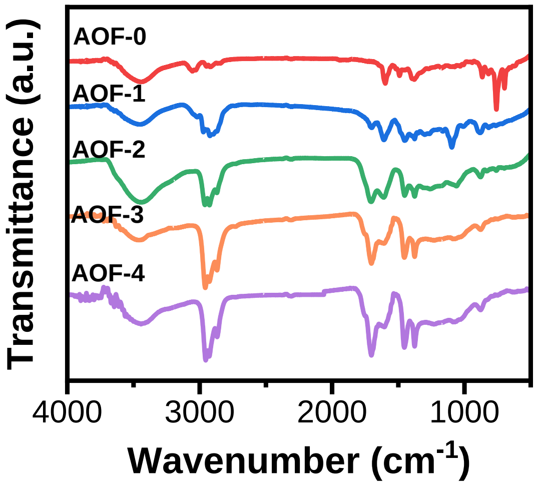

Figure S9. FT-IR spectra of different samples.

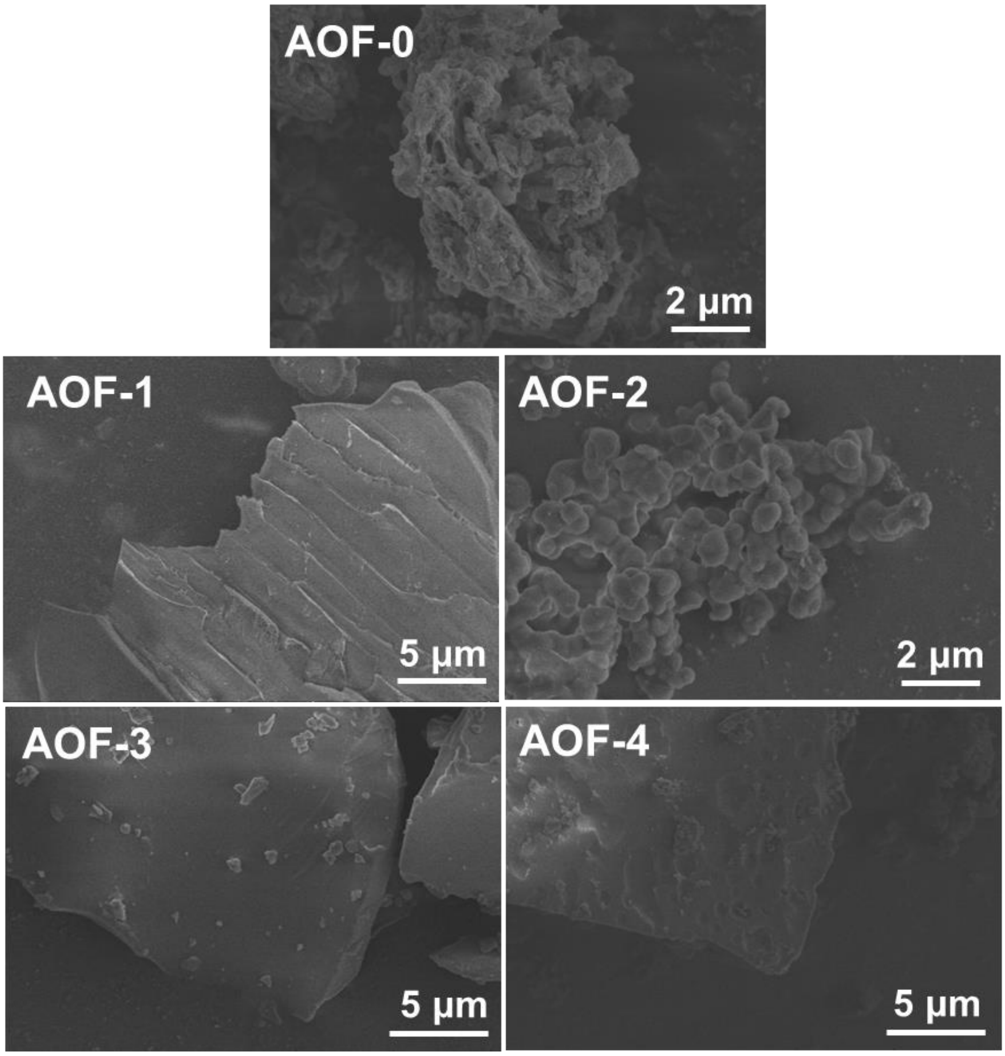

Figure S10. SEM images of different samples.

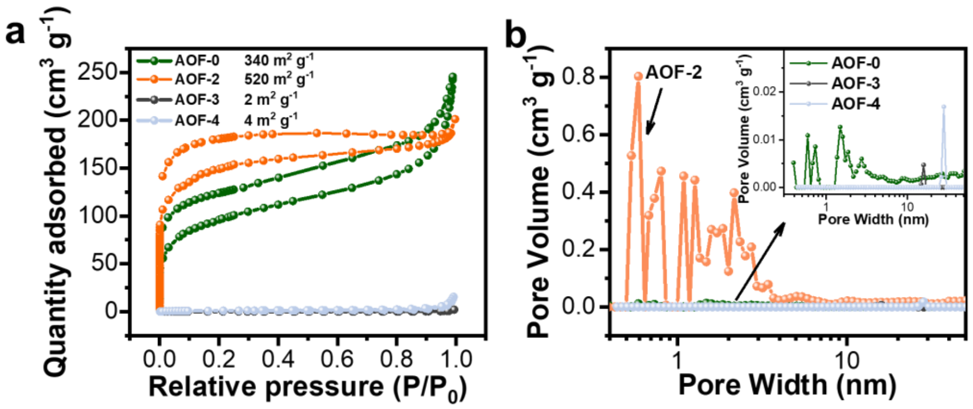

Figure S11. (a) N_2 adsorption–desorption isotherms and (b) pore-size distributions of AOF-n (n=0, 2, 3, 4).

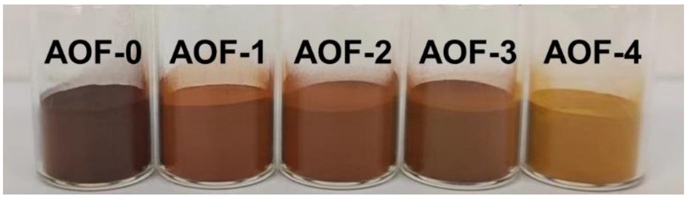

Figure S12. Photographs of different samples.

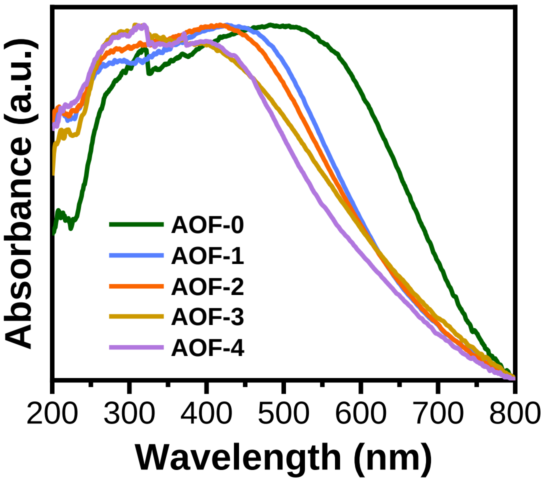

Figure S13. UV–Vis diffuse reflectance spectra of different samples.

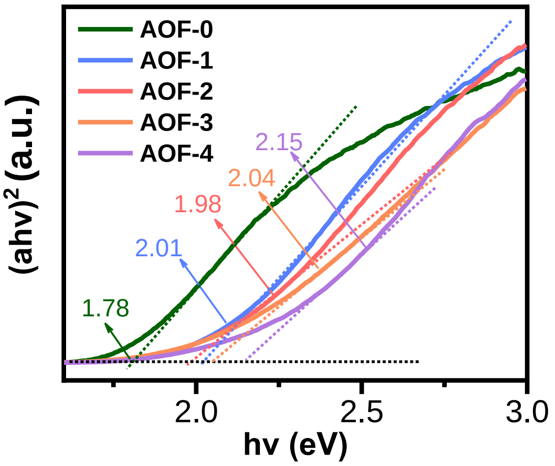

Figure S14. Tauc plot calculating the optical band gap of different samples.

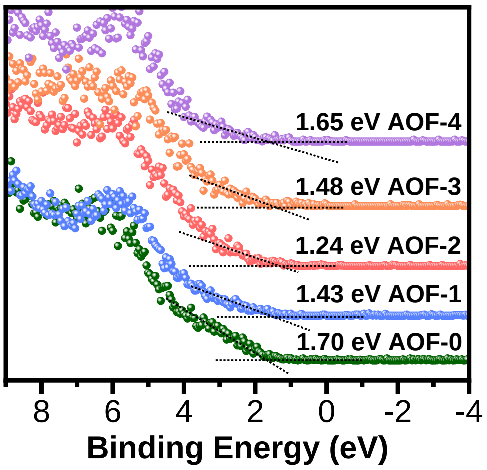

Figure S15. Valence-band XPS spectra of different samples.

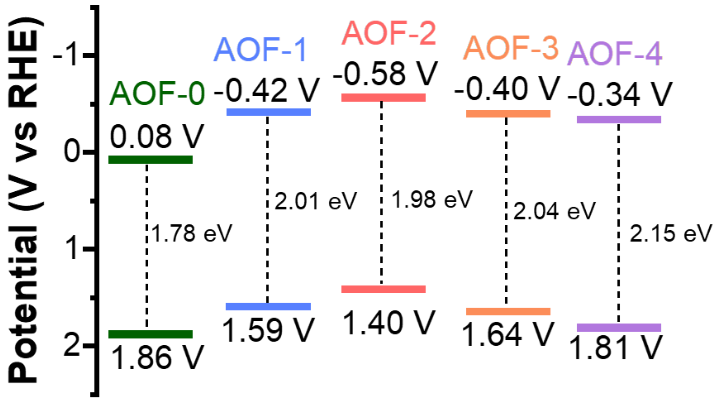

Figure S16. Energy band diagrams of different samples.

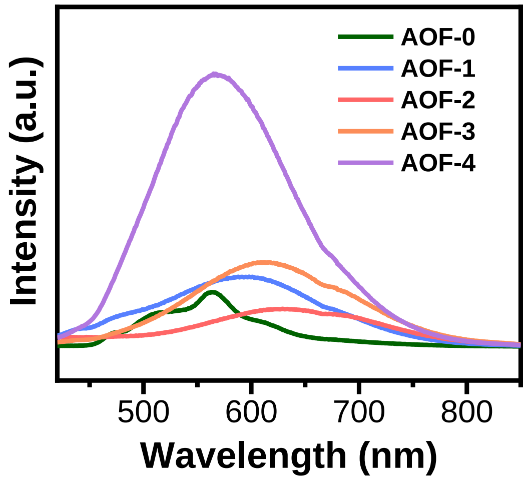

Figure S17. PL emission spectra of different samples.

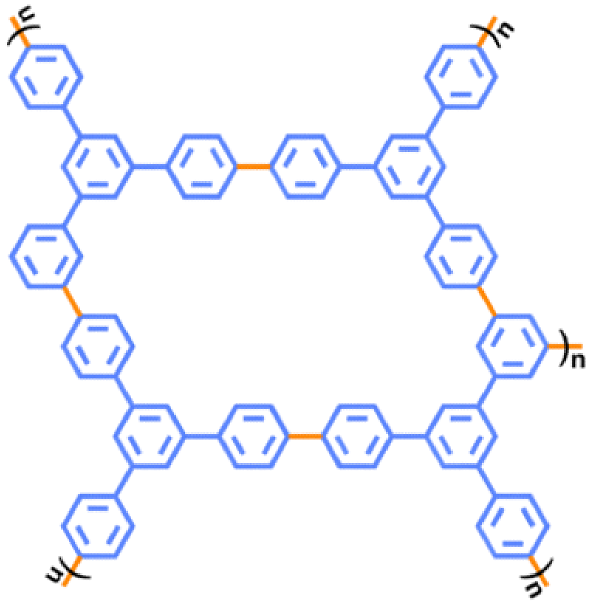

Figure S18. The possible structural model of AOF-0 prepared by Scholl Reaction.

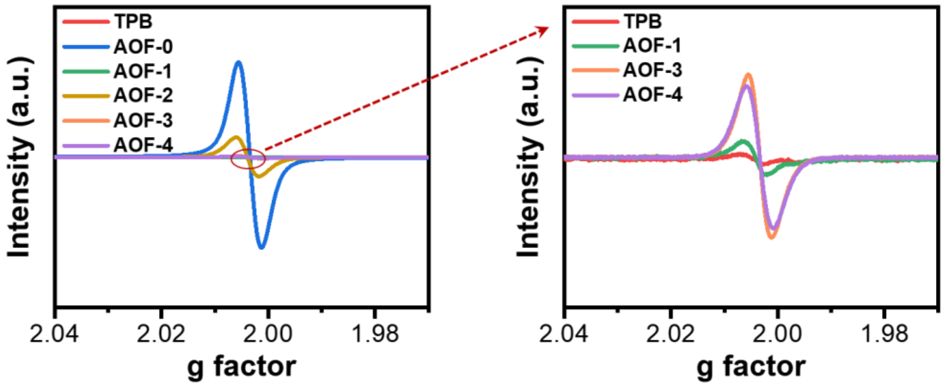

Figure S19. EPR spectra of different samples.

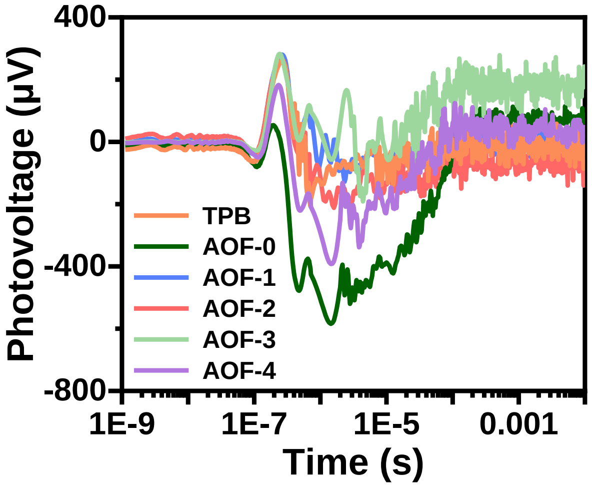

Figure S20. TPV signals of different samples.

Photocatalytic H_2O_2 decomposition over AOF-1

The AOF-1 exhibit an ultralow H_2O_2 decomposition rate. Under visible light irradiation, the concentrations of H_2O_2 could remain over 92% under 6 h visible light irradiation (Figure S24). Generally, the final amount of H_2O_2 depends on the rate of formation (K_f) and decomposition (K_d) of H_2O_2 over the catalyst. The K_f and K_d were calculated to be 45.05 μmol h^{-1} and 0.013 h^{-1} for AOF-1 (Figure 3e), indicating that AOF-1 have good ability to produce H_2O_2 and inhibit the subsequent decomposition of H_2O_2.

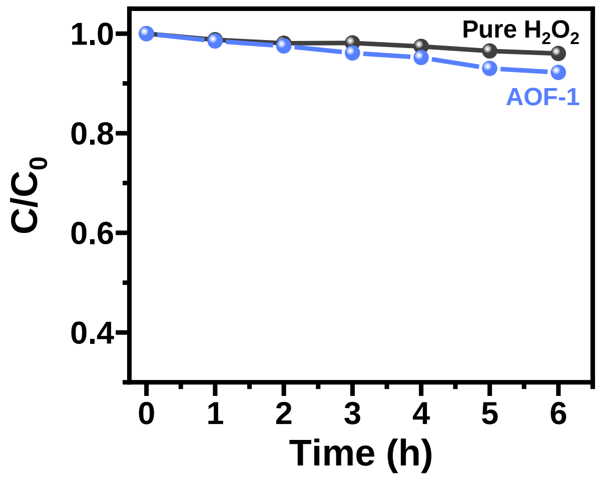

Figure S21. The photocatalytic decomposition of H_2O_2 (2 mM) under visible light irradiation.

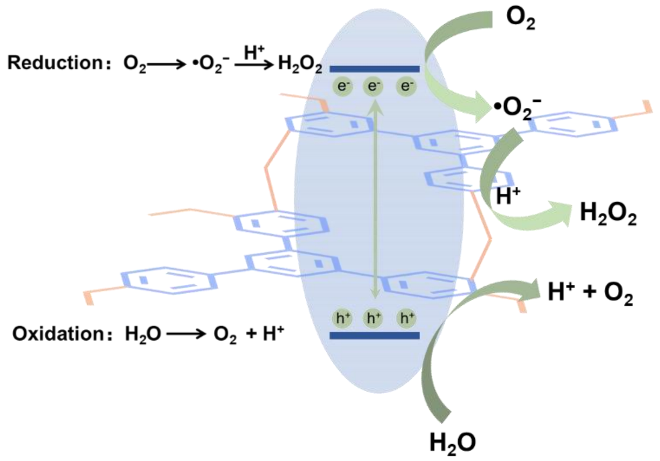

Figure S22. Proposed H_2O_2 generation mechanism over AOF-1.

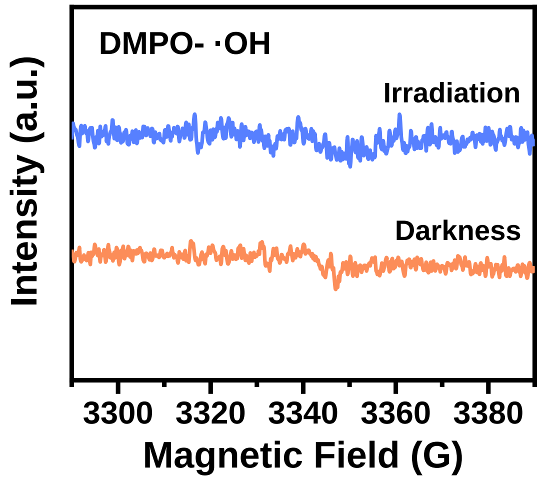

Figure S23. EPR spectra of DMPO-•OH for AOF-1.

Table S2. Photocatalytic aerobic coupling of different amines over AOF-1[a].

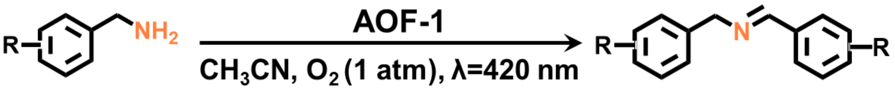

Entry	Substrate	Product	*Con.* (%)	*Sel.* (%)
1	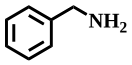	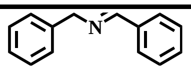	94.3 (2.5 h)	98.3
2	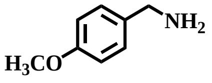	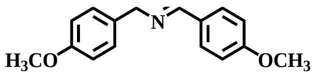	95.8 (2.5 h)	99.7
3	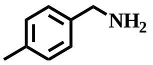	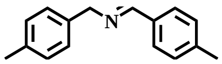	95.3 (2.5 h)	94.2
4	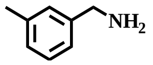	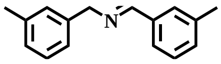	97.1 (2.5 h)	91.1
5	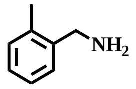	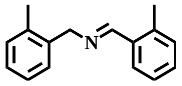	97.2 (2.5 h)	94.0
6	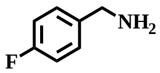	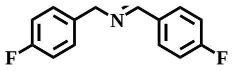	98.2 (2.5 h)	93.9
7	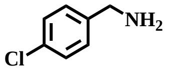	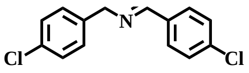	96.3 (2.5 h)	94.8
8	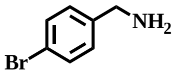	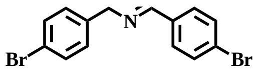	95.6 (2 h)	87.0
9	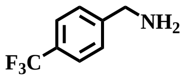	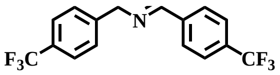	98.0 (2.5 h)	99.9
10	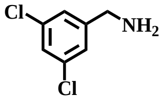	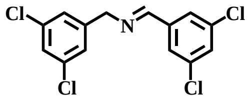	96.8 (2.5h)	92.1
11	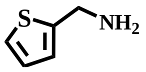	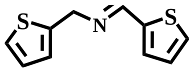	94.5 (1 h)	96.6
12	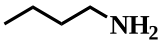	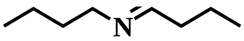	10.1 (5 h)	99.9

[a]Reaction conditions: substrate (0.5 mmol), catalyst (50 mg), O_2 (1 atm), CH_3CN (10 mL), UV LED lamp (420 nm, 90 W), temperature (298 K).

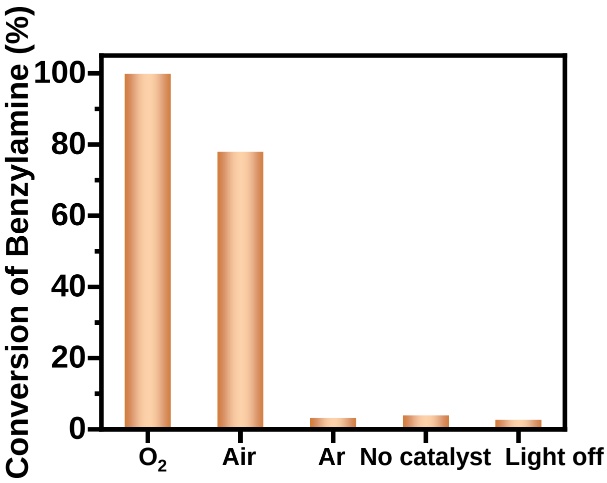

Figure S24. Photocatalytic oxidation activity of benzylamine over AOF-1 under different experimental conditions.

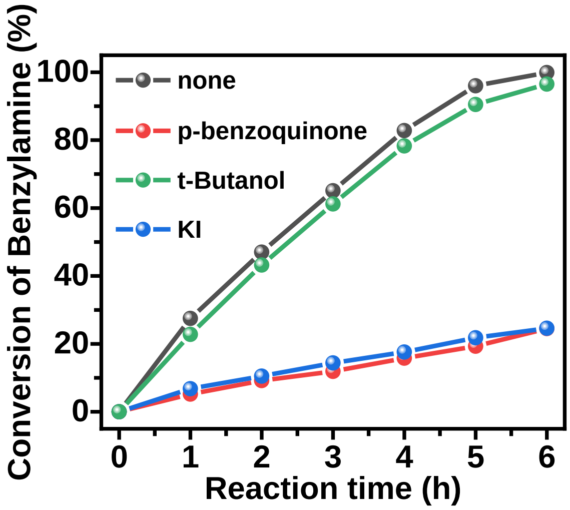

Figure S25. Oxidative coupling of benzylamine with scavengers. Reaction conditions: benzylamine (1 mmol), catalyst (50 mg), O_2 (1 atm), CH_3CN (10 mL), scavengers (1mmol), UV LED lamp (420 nm, 90 W), temperature (298 K).

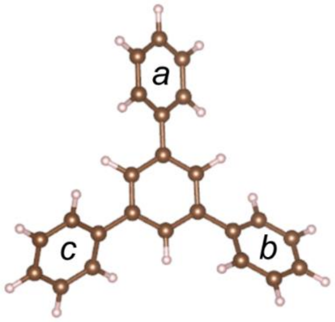

Table S3. The calculated HOMO-LUMO gap of TPB molecules with different conformations.[a]

Configuration (|*a*| |*b*| |*c*|)	Gap (eV)	Configuration (|*a*| |*b*| |*c*|)	Gap (eV)
TPB (38.05 39.11 38.48)	5.10	TPB-21 (39.44 37.54 39.02)	5.10
TPB-1 (00.00 39.11 38.05)	4.83	TPB-22 (37.74 30.50 46.95)	4.98
TPB-2 (00.00 00.00 00.00)	4.68	TPB-23 (48.96 44.86 44.87)	5.15
TPB-3 (00.00 00.00 90.00)	4.70	TPB-24 (40.28 38.61 38.61)	5.07
TPB-4 (00.00 90.00 90.00)	4.96	TPB-25 (35.56 42.61 33.92)	4.95
TPB-5 (38.05 90.00 90.00)	5.30	TPB-26 (30.90 31.68 31.69)	4.86
TPB-6 (90.00 90.00 90.00)	6.23	TPB-27 (37.30 48.39 26.37)	4.82
TPB-7 (00.00 38.05 00.00)	4.71	TPB-28 (49.33 43.67 43.68)	4.97
TPB-8 (39.11 38.02 90.00)	5.10	TPB-29 (41.77 44.92 39.48)	4.80
TPB-9 (00.00 00.00 45.00)	4.72	TPB-30 (42.75 39.77 39.78)	4.87
TPB-10 (00.00 00.00 60.00)	4.71	TPB-31 (34.81 41.21 36.07)	4.78
TPB-11 (00.00 00.00 75.00)	4.70	TPB-32 (32.39 44.62 44.62)	5.05
TPB-12 (00.00 00.00 1.15)	4.69	TPB-33 (28.53 34.94 33.75)	4.87
TPB-13 (00.00 01.16 01.15)	4.69	TPB-34 (39.33 30.05 46.50)	4.97
TPB-14 (01.16 01.16 01.15)	4.70	TPB-35 (38.05 38.48 60.18)	5.12
TPB-15 (40.61 40.30 39.69)	5.09	TPB-36 (38.05 60.08 60.18)	5.23
TPB-16 (47.91 39.05 38.77)	5.14	TPB-37 (60.09 60.08 60.18)	5.53
TPB-17 (28.60 38.79 37.91)	4.88	TPB-38 (38.60 39.11 38.48)	5.10
TPB-18 (38.62 32.50 19.89)	4.98	TPB-39 (28.06 60.18 60.08)	5.22
TPB-19 (35.01 45.25 47.37)	5.12	TPB-40 (60.18 60.18 60.08)	5.55
TPB-20 (39.77 37.24 40.47)	5.08		

[a] a, b and c represent the angles between the surrounding benzene ring and the plane, respectively.

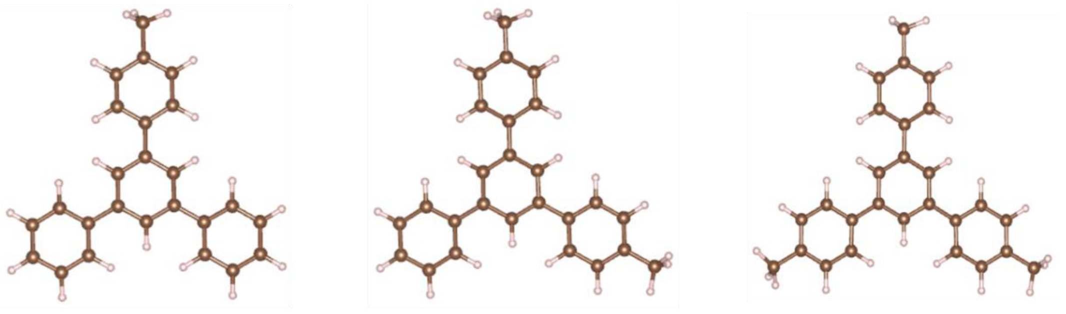

TPB-CH$_3$-1 **TPB-CH$_3$-2** **TPB-CH$_3$-3**

Figure S26. The structure of methyl substituted TPB.

Table S4. The calculated bandgap of methyl substituted TPB molecules with corresponding conformations.

Simulated model	Gap (eV)
TPB	5.10
TPB-CH_3-1	4.59
TPB-CH_3-2	4.57

TPB-CH_3-3	4.60

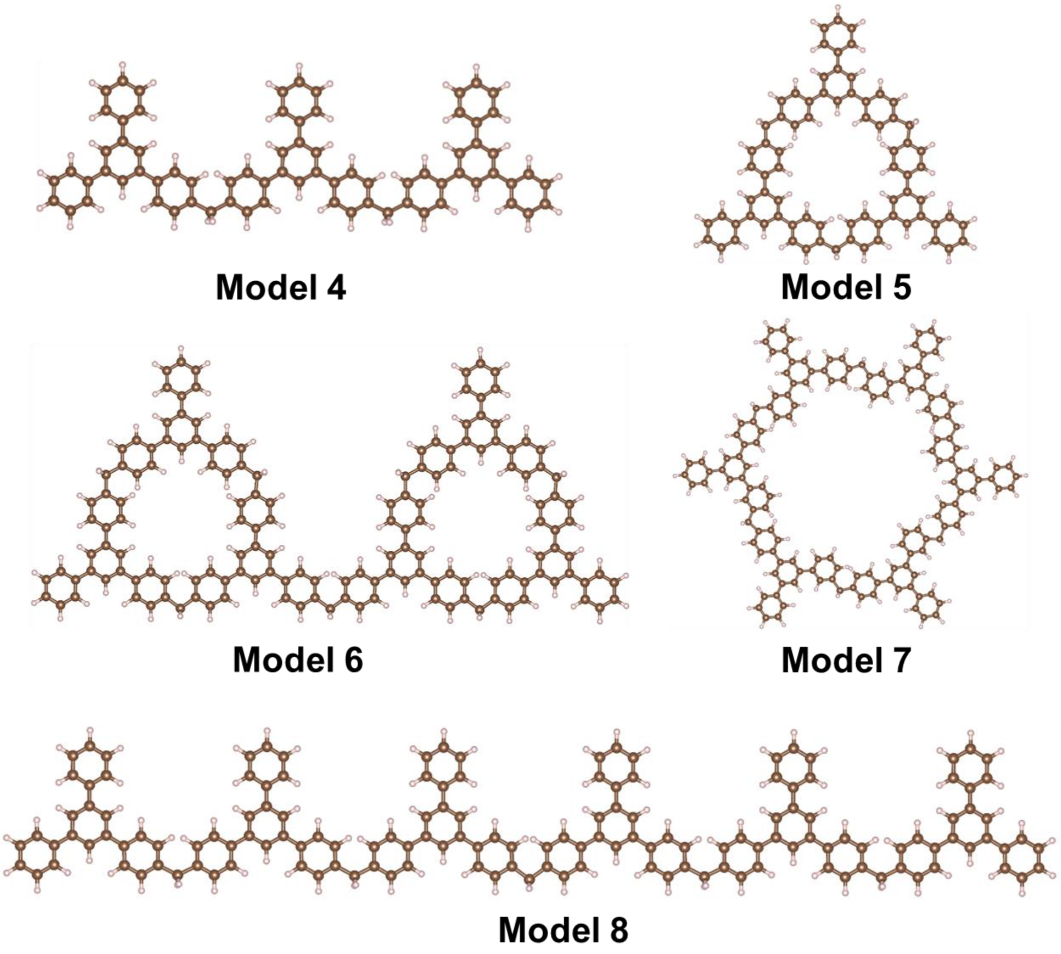

Figure S27. The structure of planar trimers and hexamers formed through TPB polymerization.

Table S5. The calculated bandgap of different model.

Simulated model	Gap (eV)
TPB	5.10
Model 4	4.53
Model 5	4.51
Model 6	4.49
Model 7	4.28
Model 8	4.51

3. References

[1] Y. Y. Li, Q. N. Wu, Q. J. Bu, K. Zhang, Y. H. Lin, D. J. Wang, X. X. Zou, T. F. Xie, *Chin. J. Catal.* **2021**, *42*, 762-771.

[2] Y. Shiraishi, S. Kanazawa, Y. Kofuji, H. Sakamoto, S. Ichikawa, S. Tanaka, T. Hirai, *Angew. Chem. Int. Ed.* **2014**, *53*, 13454-13459.

[3] Y. Kofuji, S. Ohkita, Y. Shiraishi, H. Sakamoto, S. Tanaka, S. Ichikawa, T. Hirai, *ACS Catal.* **2016**, *6*, 7021-7029.

[4] Y. Kofuji, Y. Isobe, Y. Shiraishi, H. Sakamoto, S. Tanaka, S. Ichikawa, T. Hirai, *J. Am. Chem. Soc.* **2016**, *138*, 10019-10025.

[5] Y. Kofuji, S. Ohkita, Y. Shiraishi, H. Sakamoto, S. Ichikawa, S. Tanaka, T. Hirai, *ACS Sustain. Chem. Eng.* **2017**, *5*, 6478-6485.

[6] Y. Kofuji, Y. Isobe, Y. Shiraishi, H. Sakamoto, S. Ichikawa, S. Tanaka, T. Hirai, *ChemCatChem* **2018**, *10*, 2070-2077.

[7] Y. Shiraishi, T. Takii, T. Hagi, S. Mori, Y. Kofuji, Y. Kitagawa, S. Tanaka, S. Ichikawa, T. Hirai, *Nat. Mater.* **2019**, *18*, 985-993.

[8] L. Chen, L. Wang, Y. Y. Wan, Y. Zhang, Z. M. Qi, X. J. Wu, H. X. Xu, *Adv. Mater.* **2020**, *32*, e1904433.

[9] C. H. Chu, Q. H. Zhu, Z. H. Pan, S. Gupta, D. H. Huang, Y. H. Du, S. H. Weon, Y. S. Wu, C. Muhich, E. Stavitski, K. Domen, J. H. Kima, *PNAS* **2020**, *117*, 6376-6382.

[10] Y. X. Yea, J. Pana, F. Y. Xie, L. Gong, S. M. Huang, Z. F. Ked, F. Zhua, J. Q. Xua, G. F. Ouyang, *PNAS* **2021**, *118*, e2103964118.

[11] Y. Shiraishi, M. Matsumoto, S. Ichikawa, S. Tanaka, T. Hirai, *J. Am. Chem. Soc.* **2021**, *143*, 12590-12599.

[12] S. Zhao, X. Zhao, H. Zhang, J. Li, Y. F. Zhu, *Nano Energy* **2017**, *35*, 405-414.

[13] Z. D. Zhu, H. H. Pan, M. Murugananthan, J. Y. Gong, Y. R. Zhang, *Appl. Catal. B Environ.* **2018**, *232*, 19-25.

[14] J. N. Chang, Q. Li, J. W. Shi, M. Zhang, L. Zhang, S. Li, Y. F. Chen, S. L. Li, Y. Q. Lan, *Angew. Chem. Int. Ed.* **2023**, *62*, e202218868.

[15] M. P. Kou, Y. Y. Wang, Y. X. Xu, L. Q. Ye, Y. P. Huang, B. H. Jia, H. Li, J. Q. Ren, Y. Deng, J. H. Chen, Y. Zhou, K. Lei, L. Wang, W. Liu, H. W. Huang, T. Y. Ma, *Angew. Chem. Int. Ed.* **2022**, *61*, e202200413.

[16] C. Yang, S. J. Wan, B. C. Zhu, J. G. Yu, S. W. Cao, *Angew. Chem. Int. Ed.* **2022**, *61*, e202208438.

[17] G. D. Pan, X. S. Hou, Z. Y. Liu, C. K. Yang, J. L. Long, G. C. Huang, J. H. Bi, Y. Yu, L. Y. Li, *ACS Catal.* **2022**, *12*, 14911-14917.

[18] D. Chen, W. B. Chen, Y. T. Wu, L. Wang, X. J. Wu, H. X. Xu, L. Chen, Angew. Chem. Int. Ed. 2023, e202217479.

[19] C. Zhao, X. Y. Wang, Y. F. Yin, W. M. Tian, G. Zeng, H. T. Li, S. Ye, L. M. Wu, J. Liu, *Angew. Chem. Int. Ed.* **2022**, e202218318.

[20] J. Z. Cheng, S. J. Wan, S. W. Cao, *Angew. Chem. Int. Ed.* **2023**, *62*, e202310476.

[21] P. Das, G. Chakraborty, J. Roeser, S. Vogl, J. Rabeah, A. Thomas, *J. Am. Chem. Soc.* **2023**, *145*, 2975-2984.

[22] Y. Mou, X. D. Wu, C. C. Qin, J. Y. Chen, Y. L. Zhao, L. B. Jiang, C. Zhang, X. Z. Yuan, E. Huixiang Ang, H. Wang, *Angew. Chem. Int. Ed.* **2023**, e202309480.

[23] Z. P. Luo, X. W. Chen, Y. Y. Hu, X. Chen, W. Lin, X. F. Wu, X. C. Wang, *Angew. Chem. Int. Ed.* **2023**, *62*, e202304875.

[24] J. Y. Yue, L. P. Song, Y. F. Fan, Z. X. Pan, P. Yang, Y. Ma, Q. Xu, B. Tang, *Angew. Chem. Int. Ed.* **2023**, e202309624.

[25] Y. Luo, B. P. Zhang, C. C. Liu, D. H. Xia, X. W. Ou, Y. P. Cai, Y. Zhou, J. Jiang, B. Han, *Angew. Chem. Int. Ed.* **2023**, *62*, e202305355.

[26] W. Zhao, P. Y. Yan, B. Y. Li, M. Bahri, L. J. Liu, X. Zhou, R. Clowes, N. D. Browning, Y. Wu, J. W. Ward, A. I. Cooper, *J. Am. Chem. Soc.* **2022**, *144*, 9902-9909.

[27] C. C. Qin, X. D. Wu, L. Tang, X. H. Chen, M. Li, Y. Mou, B. Su, S. B. Wang, C. Y. Feng, J. W. Liu, X. Z. Yuan, Y. L. Zhao, H. Wang, *Nat. Commun.* **2023**, *14*, 5238.

[28] Z. M. Zhou, M. H. Sun, Y. B. Zhu, P. Z. Li, Y. R. Zhang, M. K. Wang, Y. Shen, *Appl. Catal. B. Environ*. **2023**, *334*, 122862.

[29] X. C. Zhang, J. Z. Zhang, J. Miao, X. Wen, C. Chen, B. X. Zhou, M. C. Long, *Chem. Eng. J.* **2023**, *466*, 143085.

[30] C. B. Wu, Z. Y. Teng, C. Yang, F. S. Chen, H. B. Yang, L. Wang, H. X. Xu, B. Liu, G. F. Zheng, Q. H an, *Adv. Mater.* **2022**, *34,* e2110266.
